# Decay of linkage disequilibrium within genes across HGDP-CEPH human samples: most population isolates do not show increased LD

**DOI:** 10.1186/1471-2164-10-338

**Published:** 2009-07-28

**Authors:** Elena Bosch, Hafid Laayouni, Carlos Morcillo-Suarez, Ferran Casals, Andrés Moreno-Estrada, Anna Ferrer-Admetlla, Michelle Gardner, Araceli Rosa, Arcadi Navarro, David Comas, Jan Graffelman, Francesc Calafell, Jaume Bertranpetit

**Affiliations:** 1 Institut de Biologia Evolutiva (UPF-CSIC), Departament de Ciències Experimentals i de la Salut, Universitat Pompeu Fabra, Parc de Recerca Biomèdica de Barcelona, Barcelona, Spain; 2 CIBER de Epidemiología y Salud Pública (CIBERESP), Barcelona, Spain; 3 National Institute for Bioinformatics (INB), Population Genomics Node, Parc de Recerca Biomèdica de Barcelona, Barcelona, Spain; 4 Department of Pediatric, Ste Justine Hospital Research Centre, Faculty of Medicine, University of Montreal, Montreal, Quebec H3T 1C5, Canada; 5 Institució Catalana de Recerca i Estudis Avançats (ICREA), Universitat Pompeu Fabra, Barcelona, Spain; 6 Department of Statistics and Operations Research, Universitat Politècnica de Catalunya, Barcelona, Spain

## Abstract

**Background:**

It is well known that the pattern of linkage disequilibrium varies between human populations, with remarkable geographical stratification. Indirect association studies routinely exploit linkage disequilibrium around genes, particularly in isolated populations where it is assumed to be higher. Here, we explore both the amount and the decay of linkage disequilibrium with physical distance along 211 gene regions, most of them related to complex diseases, across 39 HGDP-CEPH population samples, focusing particularly on the populations defined as isolates. Within each gene region and population we use r^2 ^between all possible single nucleotide polymorphism (SNP) pairs as a measure of linkage disequilibrium and focus on the proportion of SNP pairs with r^2 ^greater than 0.8.

**Results:**

Although the average r^2 ^was found to be significantly different both between and within continental regions, a much higher proportion of r^2 ^variance could be attributed to differences between continental regions (2.8% vs. 0.5%, respectively). Similarly, while the proportion of SNP pairs with r^2 ^> 0.8 was significantly different across continents for all distance classes, it was generally much more homogenous within continents, except in the case of Africa and the Americas. The only isolated populations with consistently higher LD in all distance classes with respect to their continent are the Kalash (Central South Asia) and the Surui (America). Moreover, isolated populations showed only slightly higher proportions of SNP pairs with r^2 ^> 0.8 per gene region than non-isolated populations in the same continent. Thus, the number of SNPs in isolated populations that need to be genotyped may be only slightly less than in non-isolates.

**Conclusion:**

The "isolated population" label by itself does not guarantee a greater genotyping efficiency in association studies, and properties other than increased linkage disequilibrium may make these populations interesting in genetic epidemiology.

## Background

Linkage disequilibrium (LD) is the non-random association between allele frequencies at two loci. Recombination rate variation is the main determinant of LD [1,2]. It has been shown that recombination is extremely heterogeneous along the genome, even at short distances, which creates intricate LD patterns. LD is also shaped by demographic forces and natural selection, and has become a tool used to infer population history [3-5] and selection [6-9]. Genome- and population-related factors, then, explain why linkage disequilibrium levels vary dramatically across the genome and among some populations. The extent of LD in non-Africans is higher than in Africans [10-12], reflecting the origin and spread of modern humans from Africa, although the difference in LD between Africans and non-Africans varies greatly across loci, with examples in which it is similar or even more pronounced in Africans [13].

Linkage disequilibrium implies correlation between loci, which means that information for untyped variants can be inferred from genotyped loci in LD with them. In recent years, LD has been exploited to the extent that it has become the cornerstone concept for research in genetic epidemiology of complex diseases, since it allows indirect association mapping, as implemented in the recent flurry of genomewide association studies [14]. It is also the main justification for the HapMap project, in which single nucleotide polymorphisms (SNPs) were initially validated and genotyped at high density in four human populations [15]. The International HapMap project created a genome-wide map of LD and common haplotypes in four populations of African, European and Asian ancestry, which has been extended to eleven populations (HapMap3). Within each population, sets of reference markers tagging common haplotypes (haplotype tagSNPs or htSNPs) can be estimated, thus providing a powerful shortcut to carry out LD-based association studies. Variation in LD amount and LD patterns across human populations, though, may contribute to the notoriously poor record in replicability of association studies conducted with few SNPs [16-18].

It has often been suggested that genetically isolated populations would offer increased statistical power to detect association because of the impact of their particular past demography on their genomic structure [19]. LD in isolates would be higher than in other populations because of reduced effective population size, which limits the opportunity for recombination to erode LD. While many populations have been proposed as isolated and ideal for association studies, empirical data that verify the assumptions mentioned above are scarce. Some studies using microsatellites in the X chromosome found increased LD in the Saami from northern Scandinavia [19], which was later confirmed with SNPs [20]. In another study, Service et al. [21] compared LD levels in various genetic isolates and with an outbred European-derived sample; against that reference, most but not all isolates showed increased LD. In the latter study, while a very recent isolate created by a few founders, the Kuusamo in Finland, showed noticeably increased LD, this was not the case for other populations traditionally regarded as isolates such as the Azorean [21]. The most comprehensive SNP-based study of LD and genetic heterogeneity on an isolated population was performed on the Micronesian Kosrae, where indeed heterogeneity had decreased and LD decayed more slowly with physical distance [22].

Here we present data for 2 380 SNPs distributed across 211 gene regions in 39 worldwide populations representing human diversity (HGDP-CEPH Human Genome Cell Line Diversity Panel [23]). Gene regions were selected because they contained one or more genes of interest mostly related to complex disease. We are interested in LD in relation to genetic association studies, in which the redundancy of information implied by LD can be used to optimize genotyping. Thus, we generally did not approach LD in the genome, but in and around genes, where most variation related to disease presumably lies. Note that larger data sets of worldwide SNP variation exist [24,25], but they consist of SNP sets in commercial arrays that were selected because of their tagSNP status in specific populations [26] and, therefore, cannot be used to describe unbiased LD patterns or to detect population differences in LD.

Within each gene region and for each population we have quantified the extent of LD between all pairs of SNPs by means of r^2 ^[27] and determined the decay of LD with physical distance. Given their potential as efficient tagSNPs, we have also tallied the proportion of SNP pairs with r^2 ^larger than 0.8 in different physical distance classes and the overall per gene region. Our gene-centered approach provides an empirical view of the amount and pattern of LD decay with physical distance across worldwide human populations, including some considered as genetic isolates.

## Results

**Figure 1:**
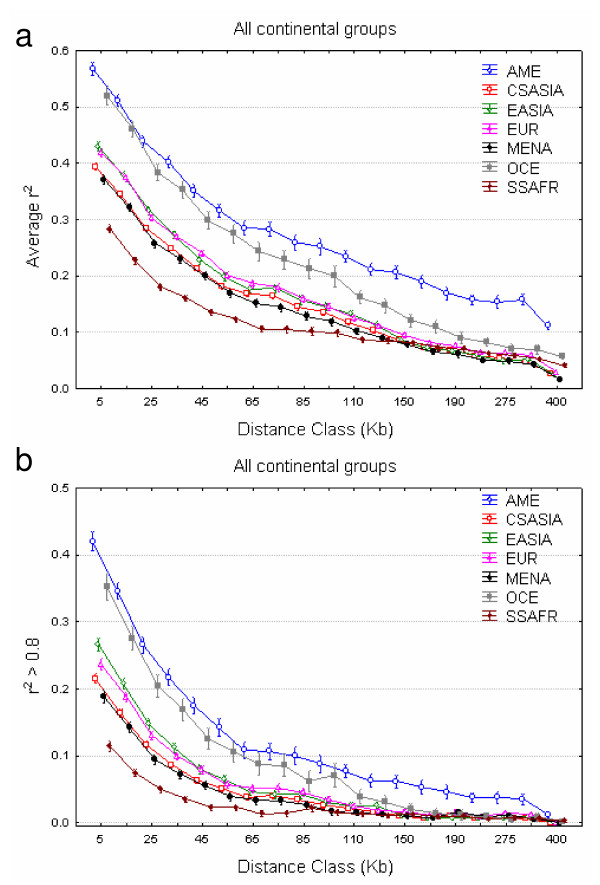
**Continental decay of linkage disequilibrium**. (A) Mean r^2 ^between all possible SNP pairs within each gene region with MAF greater than 0.05 is plotted at the midpoint of each distance class. The X-axis is not to scale. (B) The proportion of SNP pairs with r^2 ^greater than 0.8 is plotted at the midpoint of each distance class. Vertical lines represent 95% confidence intervals. The X-axis is not to scale. Continental regions are abbreviated as follows: Sub-Saharan Africa (SSAFR; including Bantu, Biaka Pygmies, Mandenka, Mbuti Pygmies, San, and Yoruba), Middle East-North Africa (MENA; including Bedouin, Druze, Mozabite, and Palestinian), Europe (EUR; including Adygei, Basque, French, North Italy, Orcadian, Russian, and Sardinian), Central South Asia (CSASIA; including Balochi, Brahui, Burusho, Hazara, Kalash, Makrani, North West China, Pathan, and Sindhi), East Asia (EASIA; including Cambodian, Han, Japanese, North East China, South China, and Yakut), Oceania (OCE; including NAN Melanesian and Papuan) and America (AME; including Colombian, Karitiana, Maya, Pima, and Surui).

The mean r^2 ^between all possible SNP pairs within each gene region and with minor allele frequencies greater than 0.05 was computed for each population and continental region and plotted against physical distance (Figure 1a and Additional file 1). Populations included in each continental region are listed in the legend of Figure 1. The amount of LD was found to be much smaller in Sub-Saharan Africa than in any other continental region; and, in accordance with their demographic histories, Oceania and America displayed greater LD at longer distances. In order to test for the effects of continental region and population affiliation within each continental group on r^2 ^variation we performed a nested ANCOVA model (see Materials and Methods). Results of the ANCOVA test show that the model and all of its components (continental group, population and physical distance class) are highly significant (p < 0.001). The amount of variation explained by the whole model was 14%; physical distance explains most of this (11%); while the continent and population within continent variables account for the remaining 2.8% and 0.5%, respectively. All mean r^2 ^pairwise comparisons between continental regions were significant (after applying the Bonferroni correction for multiple comparisons), which means that significance could not be attributed to a single continent. When we compared average LD within continental regions, the proportion of pairwise population comparisons with significant differences (after applying the Bonferroni correction post-hoc) varies greatly among regions. In Europe only 6 out of 21 (28%) pairwise comparisons were significant and all of these six pairs contained the Orcadian population. In Central South Asia, the number of statistically significant comparisons was 15 out of 36 (41%) corresponding to the Brahui and Kalash populations. For the remaining continental groups, most comparisons (~80%) were significant: 11 out of 15 pairwise comparisons between Sub-Saharan African populations, 5 out of 6 in the Middle East – North Africa, 13 out of 15 in East Asia, and 8 out of 10 in the Americas. A similar pattern of significance was observed when comparing each continental and populational mean r^2 ^value against the mean r^2 ^across continents and across populations within each continent, respectively (data not shown).

**Figure 2:**
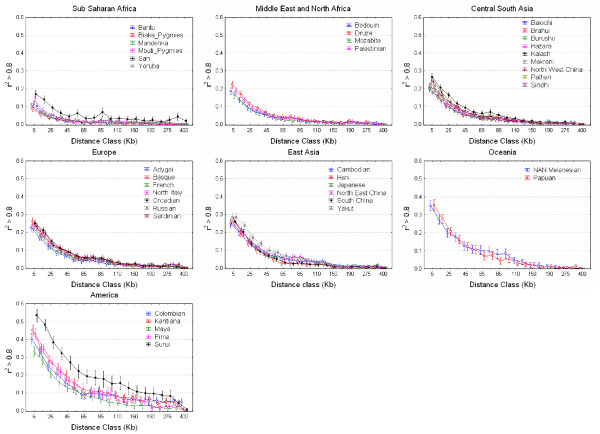
**Populational decay of linkage disequilibrium**. For each continental region, the proportion of SNP pairs with r^2 ^greater than 0.8 is plotted by distance class and population. The X-axis is not to scale.

The proportion of SNP pairs with r^2 ^greater than 0.8 was counted for each population and continental region and plotted against physical distance (Figure 1b and Figure 2). The average proportion of SNP pairs with r^2 ^> 0.8 per gene region varied from ~3% in Sub-Saharan Africans to ~17% in Americans, which illustrates the differences in LD among continental groups and the different efficiencies of tagging SNP strategies across continents. In general, the proportion of SNPs with r^2 ^> 0.8 was found to be significantly different across continental regions for all physical distance classes (with the smallest *χ*^2 ^test value equal to 126.63, 6 df, p < 10^-8^). In contrast, comparisons within continents are generally not significant, with a few exceptions. Sub-Saharan Africans (with the small San sample removed) were significantly different (p < 0.05) in their proportion of SNP pairs with r^2 ^> 0.8 for only two distance classes (0–10 and 250–300 kb), which were not significant after Bonferroni correction. The Middle East was significant in the first four distance classes (the first two after Bonferroni correction); this was also the case for seven distance classes in Central Asia (four after Bonferroni correction). European populations were heterogeneous for the 10–20, 30–40, and 300–400 kb distance classes, albeit none of these comparisons would be significant after Bonferroni correction. The number of heterogeneous distance classes in East Asia was five and two (60–70 and 70–80 kb) before and after Bonferroni correction, respectively. Finally, Americans were heterogeneous at all distance bins, and this effect cannot be attributed to any single population: dropping the apparently more differentiated population, the Surui, still results in 14 significant tests in 19 distance bins.

Several populations of the HGDP-CEPH panel are cultural, linguistic, or demographic isolates (Additional file 2). To test whether such isolation has a significant impact in practical terms on LD, we counted the number of SNP pairs with r^2 ^> 0.8 per gene region. In Sub-Saharan African populations, we compared the two Pygmy samples against the non-isolated Mandenka, Bantu, and Yoruba. In the Pygmy samples, the proportion of SNP pairs with r^2 ^> 0.8 per gene region was only slightly lower than in non-Pygmies: 2.8% vs. 2.9% (*χ*^2 ^= 0.351, p = 0.562). In the Middle East and North Africa the only non-isolated population are the Palestinians; however, the proportion of SNP pairs with r^2 ^> 0.8 per gene region was not significantly different between Palestinians and the three isolated populations (Mozabites, Bedouins and Druzes). In the Central South Asian samples we compared the Kalash with all other Pakistani populations pooled together. We found significantly higher proportions of SNP pairs with r^2 ^> 0.8 in the Kalash (9.0% vs. 6.5%, *χ*^2 ^= 149, p < 10^-8^). In Europe, after dividing the populations in isolates (Orcadians, Sardinians and Basques) vs. non isolates (French, North Italians, Adygei and Russians) high-LD SNP pairs were 8.5% in isolates vs. 8.1% in non-isolates (*χ*^2 ^= 8.773, p = 0.003). In East Asia, we compared the Yakut against all other populations and found a slightly higher increase (10.1% vs. 8.4%, *χ*^2 ^= 45.31, p < 0.001). Finally, in the Americas we compared the Surui vs. the rest and found the largest difference (24.8% vs. 15.2%, *χ*^2 ^= 477, p < 10^-8^). In summary, we found that the proportion of SNP pairs with r^2 ^> 0.8 was significantly higher in population isolates as compared to non-isolates in some but not all continental regions. Moreover, when analyzing the proportion of SNP pairs with r^2 ^> 0.8 by physical distance classes between isolated and non-isolated populations within each continent, only the Kalash and the Surui displayed statistically significant larger proportions consistently for several distance bins after Bonferroni correction. It may be argued that removing SNPs with MAF < 0.05 populationwise would bias the results by dropping more SNPs in isolated populations; however, the proportion of polymorphic SNPs removed in African isolates (11.2%) was just slightly higher than in non-isolates (8.8%), as was also the case in East Asia (9.8% vs. 8.2%), while in Europe, the Middle East/North Africa, and Central South Asia, the difference was negligible (4.5% vs. 4.1%, 3.7% vs. 3.6%, and 5.1% vs. 4.7%, respectively).

## Discussion

The amount of LD and the proportion of SNPs with r^2 ^> 0.8 showed similar patterns: heterogeneity among continental regions and higher homogeneity among populations within each geographical region, with the exception of the Americas. Although correlated, these two measures of overall LD capture different aspects: mean r^2 ^offers a broad picture of LD, while the proportion of r^2 ^values > 0.8 focuses on the higher end of the LD spectrum, where information redundancy between SNPs is higher to the point that it is the usual threshold where tagSNPs are designed. We have confirmed the previously observed trend of an LD decline from Sub-Saharan Africa (with the lowest levels of LD) to successively increasing amounts of LD in Middle East-North Africa, Central South Asia, Europe, East Asia, Oceania and America (with the highest amount of LD) [10,12]. Previous observations were based either on a few genes and a similar geographical range of samples [10,12] or, on the contrary, on a higher number of markers limited to a small number of populations, such as the three HapMap or Perlegen populations [15,28]. A basic description of LD decline with distance has been published for a subset of the HGDP-CEPH panel for > 500 000 SNPs [25], outlining the general trends that we have analyzed in detail. We have explored the LD patterns in 39 worldwide populations by means of the r^2 ^measure of LD between 21 685 SNPs pairs covering 211 autosomal gene regions. Moreover, we have specifically focused on those SNP pairs with high LD (so that each one can be used to tag the other) in a relevant subset of genes, as most of them may be implicated in common diseases. Note that our results may not be applied to the whole genome, but they are highly relevant to candidate-gene association studies. In this context, we have extended the previous observation that more tagSNPs are needed in the Yoruba than in Europeans or Asians [29] to a wider range of African populations that show similarly low levels of LD. On average, 3.14% of the SNP pairs in our study showed r^2 ^> 0.8 in Sub-Saharan Africans vs. 7.52% in Europeans; that is, 2.4 times as many SNP pairs showed high levels of information redundancy in Europeans than in Africans. We found a general pattern of greater LD differences between continents than within them, which would imply that genetic association studies should be more easily replicated within than between continents, as previously indicated in a set of dopamine and serotonin pathway genes [30]. Similarly, our results point to a high transferability of tagSNPs within continents [31], with the exception of America. This pattern reflects the extremely heterogeneous nature of the American populations as reflected, for instance, in their STR allele frequencies [32]. Apparently, after the bottleneck associated with the first colonization of the Americas, which increased LD, genetic drift has acted extensively to differentiate American populations in their allele and haplotype frequencies as well as in their levels of LD.

A role has been suggested for genetically isolated populations in genetic epidemiology because of their predicted high levels of LD, which would facilitate the detection of genes involved in complex diseases by indirect association [19]. In the HGDP-CEPH panel, several populations can be considered as cultural and genetic isolates (Additional file 2). Such populations showed moderate increases in the proportion of SNP pairs with r^2 ^> 0.8 per gene region when compared with the non-isolates in their respective continents. Conversely, if we take the proportion of SNP pairs with r^2 ^< 0.8 as a rough indication of the minimum proportion of SNPs that are needed to capture the haplotype variation in a gene region, then the difference in the number of the SNPs that need to be typed in isolated populations compared to their non-isolate continental counterparts would be of 0.4% in the European isolates, 1.8% in the Yakut, 2.7% in the Kalash, and 11.3% in the Surui. Thus, genotyping costs may be slightly more economical in isolated than in outbred populations. However, association studies designed in the latter may have two practical advantatges: i) possibly larger sample sizes can be obtained in general populations, and ii) allele frequencies may be closer to those in reference HapMap populations, which allows more precise a priori statistical power calculations and prevents genotyping SNPs that can result monomorphic.

It follows, then, that being labelled a population isolate by genetic, linguistic or cultural evidence is not sufficient to harbor increased LD to a point that would justify a significant reprieve in the genotyping burden for genetic association studies. This result agrees with a separate analysis [33] in the CEPH-HGDP panel, in which the microsatellite-based estimates of the *θ *= 4N_e_*μ *paramater were not significantly lower in isolated than in mainstream populations within each continent. Considering mutation rates (*μ*) as equal across populations, it follows that effective population sizes are not detectably lower in population isolates. A presumably reduced effective population size is indeed the condition that would increase LD in isolated populations. The levels of isolation required to decrease Ne significantly and subsequently increase LD appear to have been rare in the human demographic history, at least in the populations sampled for the CEPH-HGDP panel. Examples of isolated populations with significantly increased LD are the Kuusamo Finns [21] and the Micronesian Kosrae [22]; in the CEPH-HGDP panel, the only isolated populations with consistently increased LD in all distance classes with respect to their continent are the Kalash (Central South Asia) and the Surui (America). The Kalash were noticed as an outlier for their allele frequencies in 377 STRs [34], although a more recent survey of 642 690 SNPs failed to replicate this finding [24]; the Surui, even though all presumed related individuals have been dropped from the analysis, may share many recent common ancestors [35].

The present study was designed to mimic the conditions under which most genomewide association studies are performed, namely: i) focus on gene regions; ii) common SNPs, usually defined through a MAF threshold in a reference population, and iii) use of tagSNPs, often defined with a r^2 ^> 0.8 threshold. We have shown that, under these conditions, the SNPs that would be needed to be typed are just slightly less in isolates. It is increasingly being recognized, and we provide empirical results to that effect, that the value of isolated populations in genetic epidemiology lies not in their higher LD brought about by a presumably reduced Ne, but due to other characteristics such as large and accessible families, deep genealogical records or a low environmental variance.

## Conclusion

We have explored both the levels and decay of LD with physical distance along 211 gene regions mostly related to complex disease across 39 worldwide human populations. When focusing on the populations considered to be isolates, the main result of our gene-centered approach is that these isolates do not usually show increased levels of LD as measured by the proportion of SNPs with r^2 ^greater than 0.8. These results led us to conclude that the "isolated population" label by itself does not guarantee a greater genotyping efficiency in association studies, and that properties other than increased LD may make these populations interesting in genetic epidemiology.

## Methods

### SNPs

We analyzed a total of 2 380 SNPs covering 211 gene regions with a mean of 11 SNPs per gene region, and a mean distance of 10.79 kb between consecutive SNPs (see Additional file 3). The median and maximum length per gene region are 73.2 and 1928 kb, respectively. We can distinguish four main functional categories in the gene regions analysed: 116 genes (792 SNPs) are involved in processes that, if disrupted, could lead to cancer; 58 genes (917 SNPs) are involved in glycosylation, pathogen recognition and/or immune response; 21 genes (376 SNPs) are involved in neurotransmission or neurodevelopment and may be implicated in psychiatric disorders; and 16 genes (295 SNPs) belong to other diverse functional categories. Preference was given to SNPs with an *a priori *minor allele frequency (MAF) over 0.10, which were compiled from HapMap and dbSNP databases. Additionally, coding SNPs and other functional SNPs identified using PupaSNP Finder [36] were also included for analysis when possible, regardless of their allele frequency or validation status. Note that LD or tagSNPs status were not criteria in selecting SNPs for our study. SNPs were typed using either the SNPlex (Applied Biosystems, 59.2%), the BeadArray (Illumina, 40.3%), or Taqman technologies (0.05%). The raw success rates for each genotyping technology were respectively 87.42%, 89.40% and 92.3%.

### Samples

We analysed the H971 subset of the Human Genome Diversity Cell Line Panel (HGDP-CEPH) recommended by Rosenberg [37]. The 51 original HGDP-CEPH population samples [23] were re-grouped into 39 populations based on geographic and ethnic criteria as in Gardner et al. [38] to avoid some small sample sizes. For part of the analysis, populations were further grouped into seven main geographical regions (see legend of Figure 1). Given their cultural, linguistic, demographic, or genetic distinctiveness, some populations were considered as isolates and treated separately in some analyses. These were the Biaka and Mbuti Pygmies (Sub-Saharan Africa), Mozabites, Bedouins, and Druzes (Middle East – North Africa); Orcadians, Sardinians, and Basques (Europe); the Kalash (Central South Asia), the Yakut (East Asia), and the Surui (America). In each case, appropriate evidence for isolate consideration is presented in Additional file 2.

### Data analysis

Genotype data was collected and stored in a database within the SNPator [39] web environment , where part of the analyses such as control for replicate samples and basic analysis such as allele frequencies, expected heterozygosity and Hardy-Weinberg equilibrium Chi-square tests were performed. Haplotypes were estimated using fastPHASE [40] for each gene region and population. For each population, linkage disequilibrium was measured as r^2 ^[27] for all SNP pairs within each gene region. Distances between SNP pairs were classified into bins of 0–10 kb (2 040 SNP pairs), 10–20 kb (2 231 SNP pairs), 20–30 kb (2 040 SNP pairs), 30–40 kb (1 781 SNP pairs), 40–50 kb (1 396 SNP pairs), 50–60 kb (1 156 SNP pairs), 60–70 kb (994 SNP pairs), 70–80 kb (904 SNP pairs), 80–90 kb (732 SNP pairs), 90–100 kb (687 SNP pairs), 100–120 kb (1 147 SNP pairs), 120–140 kb (944 SNP pairs), 140–160 kb (823 SNP pairs), 160–180 kb (672 SNP pairs), 180–200 kb (604 SNP pairs), 200–250 kb (1 092 SNP pairs), 250–300 kb (692 SNP pairs), 300–400 kb (909 SNP pairs), more than 400 kb (841 SNP pairs). SNPs with allele frequencies below 0.05 in a particular population were not considered for further analysis in that population. This procedure created slight differences in the number of SNP pairs between populations (see Additional file 4). Alternatively, we could have dropped SNPs not common to all populations, but then the number of SNPs would have decreased drastically.

The mean r^2 ^between all possible SNP pairs within each gene region was computed for each population and continental region. In order to achieve approximate normality, the r^2 ^variable was square-root and Box-Cox transformed (*λ *= 0.34) using the car (Companion to Applied Regression; available at ) package implemented in the R programme. A nested model of covariance analysis (ANCOVA) was applied using population as a factor nested in the continent variable. Distance class (as defined above) was treated as a covariable in order to control for the effect of physical distance on LD (as measured by r^2^). The experimental design can be written as the following linear model:



where for a couple of SNPs (within a given gene), *y*_*ijk *_is the transformed r^2 ^value, *μ *is the overall grand mean of the r^2 ^transformed value; *βX*_*ij *_is the effect explained by the physical distance class; *T*_*i *_is the effect of the *i*th continent (Sub-Saharan Africa, Middle East – North Africa, Europe, Central South Asia, East Asia, Oceania, the Americas); *R*_*j*(*i*) _is the effect of the *j*th population (Adygei, Balochi, ...) within continent *i*; and *ε*_*ijk *_is the residual error associated with the corresponding transformed r^2 ^value of the *ijk*th element. Significance of mean r^2 ^comparisons between continental regions and between populations within each region were conservatively evaluated for the whole model and using the Bonferroni correction for multiple comparisons.

The proportion of SNP pairs with r^2 ^greater than 0.8 was counted for each population and continental region. Significance of differences in the proportion of SNP pairs with r^2 ^> 0.8 across continental regions and among populations within each geographical region for all physical distance classes was evaluated through *χ*^2 ^tests; p-values were corrected for multiple testing with the conservative Bonferroni correction. The mean r^2 ^and proportion of SNP pairs with r^2 ^above 0.8 are available in Additional file 4.

## Authors' contributions

EB supervised the SNP genotyping analysis, contributed to the analysis and interpretation of data, and drafted the manuscript. HL contributed to part of the statistical analysis and to the interpretation of data. CMS contributed to part of the statistical analysis. AME, AFA, MG, and AR selected the gene regions under study, carried out the SNP genotyping analysis and carried out descriptive statistical analyses on the data. FCas, AN and DC participated in the design and coordination of the study and helped to draft the manuscript. JG contributed to the design of the study and to part of the statistical analysis. FCal participated in the design of the study, supervised the statistical analysis, contributed to the interpretation of data and helped to draft the manuscript. JB conceived the study, contributed to the interpretation of data, and helped to draft the manuscript. All authors read and approved the final manuscript.

## Supplementary Material

Additional file 1 **Populational decay of linkage disequilibrium**. For each continental region, mean r^2 ^between all possible SNP pairs within a gene region and with MAF greater than 0.05 is plotted by distance class and population. The X-axis is not to scale. Click here for file

Additional file 2 **Characteristics of population isolates**. External evidence for the cultural, linguistic, demographic, or genetic distinctiveness of the populations considered as isolates in some analyses. Abbreviations: mitochondrial DNA (mtDNA), Y (Y chromosome), Haemoglobin (Hb), Single Nucletide Polymorphisms (SNPs), Restriction Fragment Length Polymorphisms (RFLP), Short Tandem Repeats (STRs). Click here for file

Additional file 3 **List of gene regions used in this study**. SNP pairs refers to all possible SNP pairs within each gene region; mean SNP pairs is the average number of SNP pairs actually used per population after dropping those with MAF < 0.05. Abbreviations: CAN, cancer-related genes; GLY, genes involved in glycosylation; IMM, genes related to pathogen recognition and/or immune response; PSY, genes involved in neurotransmission or neurodevelopment; and others, genes belonging to other diverse functional categories. Click here for file

Additional file 4 **Linkage disequilibrium parameters for each population and distance class**. Mean r^2 ^and proportion of SNP pairs with r^2 ^> 0.8 for each population and distance class. Abbreviations: N, number of SNP pairs; 2n, maximum sample size (chromosomes). Click here for file
