# Supplementary figures and images for: Decay of linkage disequilibrium within genes across HGDP-CEPH human samples: most population isolates do not show increased LD
Source: BMC Genomics. 2009 Jul 28;10:338. doi: 10.1186/1471-2164-10-338 (PMC2723139; doi:10.1186/1471-2164-10-338)

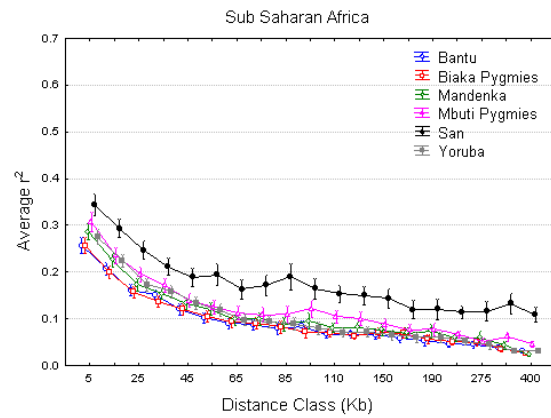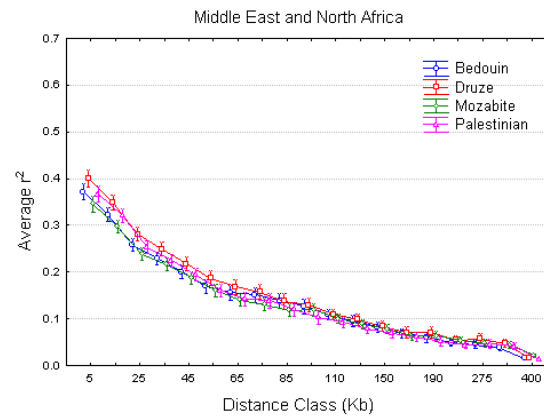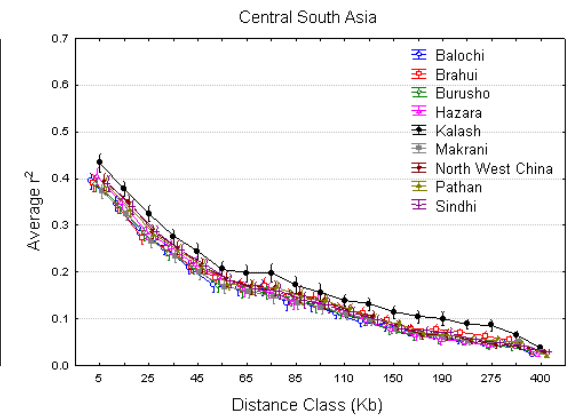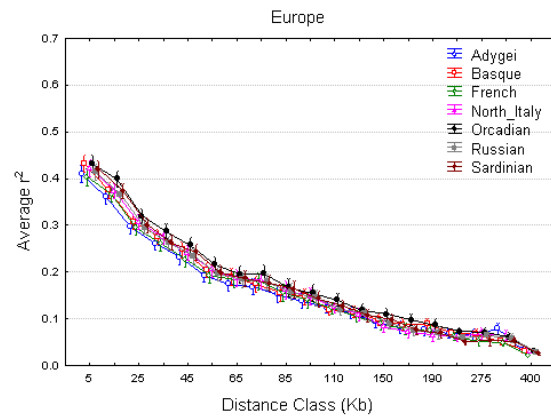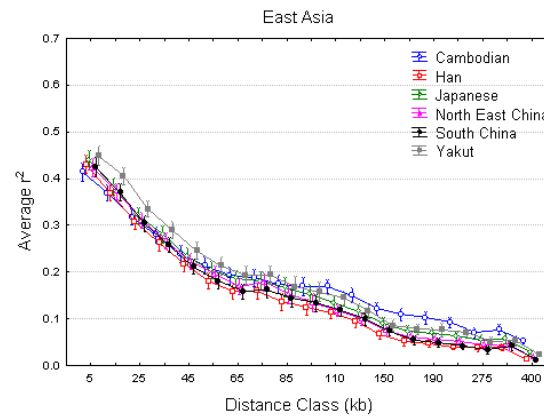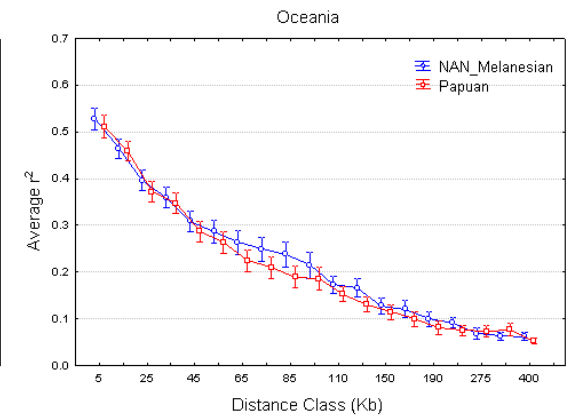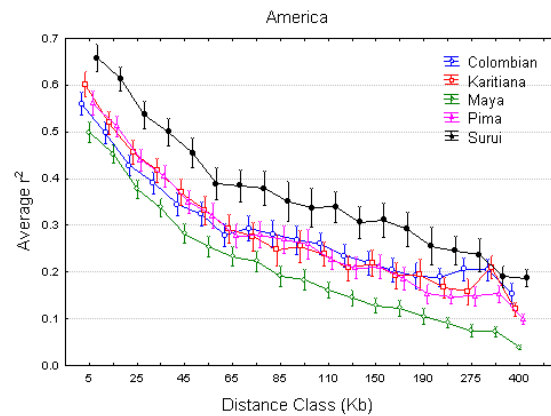

Supplement: Additional file 1 — Populational decay of linkage disequilibrium. For each continental region, mean r2 between all possible SNP pairs within a gene region and with MAF greater than 0.05 is plotted by distance class and population. The X-axis is not to scale. [file 1471-2164-10-338-S1.pdf]
