# Supplementary material for: Decay of linkage disequilibrium within genes across HGDP-CEPH human samples: most population isolates do not show increased LD
Source: BMC Genomics. 2009 Jul 28;10:338. doi: 10.1186/1471-2164-10-338 (PMC2723139; doi:10.1186/1471-2164-10-338)
Supplement: Additional file 2 — Characteristics of population isolates. External evidence for the cultural, linguistic, demographic, or genetic distinctiveness of the populations considered as isolates in some analyses. Abbreviations: mitochondrial DNA (mtDNA), Y (Y chromosome), Haemoglobin (Hb), Single Nucletide Polymorphisms (SNPs), Restriction Fragment Length Polymorphisms (RFLP), Short Tandem Repeats (STRs). [file 1471-2164-10-338-S2.doc]

**Supplementary Table 2.** Characteristics of population isolates. External evidence for the cultural, linguistic, demographic, or genetic distinctiveness of the populations considered as isolates in some analyses. Abbreviations: mitochondrial DNA (mtDNA), Y (Y chromosome), Haemoglobin (Hb), Single Nucletide Polymorphisms (SNPs), Restriction Fragment Length Polymorphisms (RFLP), Short Tandem Repeats (STRs).

| Population | Genetic | Linguistic | Demographic | Notes |
| --- | --- | --- | --- | --- |
| ***Sub-Saharan Africa*** | |  |  |  |
| Biaka and Mbuti Pygmies | [1-3](mtDNA); [4-6] (Y-chromosome) | Biaka: Aka (Bantu)  http://www.ethnologue.com/show_language.asp?code=axk  Mbuti: Lese (Nilo-Saharan)  http://www.ethnologue.com/show_language.asp?code=les | [7] | Small groups of hunter-gatherers in the rainforest of Central Africa |
| ***Middle East-North Africa*** | |  |  |  |
| Mozabites | [8](mt DNA) ; [9](Y); [10](Hb) | Tumzabt dialect (Berber) http://www.ethnologue.com/show_language.asp?code=mzb |  | Islam sect known as Kharejites; in oases of the Algerian Sahara |
| Bedouins | [11](mtDNA); [12](Y) | Arabic dialect  http://www.ethnologue.com/show_language.asp?code=ars | [13] | Semi-nomadic pastoralists; strong tribal structure based on kinship |
| Druzes | [14](mtDNA); [15](mtDNA, Y) | Arabic |  | Strictly endogamous religious sect, not considered Muslims by other Muslims |
| ***Europe*** | |  |  |  |
| Orcadians | [16](mtDNA); [17](Y); [18, 19](mtDNA, Y) | Dialect of Scots  http://www.ethnologue.com/show_language.asp?code=sco | [20] | Island population, north of Scotland; originally inhabited by Neolithic tribes |
| Sardinians | [21, 22] (mtDNA); [23](Y); [24](mtDNA, Y); [25, 26](autosomal SNPs) | Romance language  http://www.ethnologue.com/show_language.asp?code=src | [24] | Island population setled in old prehistory, Neolithic times |
| Basques | [27](mtDNA); [28](Y); [29](autosomal *classical* markers);[30] (review of autosomal disease mutations) | Language isolate  http://www.ethnologue.com/show_language.asp?code=eus |  | Westernmost part of the Pyrenees, both in France and Spain |
| ***South Asia*** | |  |  |  |
| Kalash | [31](STRs);[32](autosomal SNPs) [33](mtDNA); [34](Y) | Kalash language, Dardic group, Indo-Iranian subfamily  http://www.ethnologue.com/show_language.asp?code=kls |  | Non-muslim religious isolate in Pakistan; reduced population size (~6,000) |
| ***East Asia*** | |  |  |  |
| Yakut | [35](mtDNA, Y chr) | Yakut or Sakha language, of the Northern branch of the Turkic subfamily  http://www.ethnologue.com/show_language.asp?code=sah |  | Former hunters and reindeer herders in Yakutia (Rusia) |
| ***America*** | |  |  |  |
| Surui | [36](RFLPs), [37](STRs) | Tupi language  http://www.ethnologue.com/show_language.asp?code=sru |  | Pará region of Brazil; very small population |

**References for Supplementary Table 2**

1. Quintana-Murci L, Quach H, Harmant C, Luca F, Massonnet B, Patin E, Sica L, Mouguiama-Daouda P, Comas D, Tzur S *et al*: **Maternal traces of deep common ancestry and asymmetric gene flow between Pygmy hunter-gatherers and Bantu-speaking farmers**. *Proc Natl Acad Sci U S A* 2008, **105**(5):1596-1601.

2. Batini C, Coia V, Battaggia C, Rocha J, Pilkington MM, Spedini G, Comas D, Destro-Bisol G, Calafell F: **Phylogeography of the human mitochondrial L1c haplogroup: genetic signatures of the prehistory of Central Africa**. *Mol Phylogenet Evol* 2007, **43**(2):635-644.

3. Destro-Bisol G, Coia V, Boschi I, Verginelli F, Caglia A, Pascali V, Spedini G, Calafell F: **The analysis of variation of mtDNA hypervariable region 1 suggests that Eastern and Western Pygmies diverged before the Bantu expansion**. *Am Nat* 2004, **163**(2):212-226.

4. Destro-Bisol G, Donati F, Coia V, Boschi I, Verginelli F, Caglia A, Tofanelli S, Spedini G, Capelli C: **Variation of female and male lineages in sub-Saharan populations: the importance of sociocultural factors**. *Mol Biol Evol* 2004, **21**(9):1673-1682.

5. Kayser M, Krawczak M, Excoffier L, Dieltjes P, Corach D, Pascali V, Gehrig C, Bernini LF, Jespersen J, Bakker E *et al*: **An extensive analysis of Y-chromosomal microsatellite haplotypes in globally dispersed human populations**. *Am J Hum Genet* 2001, **68**(4):990-1018.

6. Underhill PA, Shen P, Lin AA, Jin L, Passarino G, Yang WH, Kauffman E, Bonne-Tamir B, Bertranpetit J, Francalacci P *et al*: **Y chromosome sequence variation and the history of human populations**. *Nat Genet* 2000, **26**(3):358-361.

7. Cavalli-Sforza LL: **African Pygmies**. New York: Academic Press; 1986.

8. Corte-Real HB, Macaulay VA, Richards MB, Hariti G, Issad MS, Cambon-Thomsen A, Papiha S, Bertranpetit J, Sykes BC: **Genetic diversity in the Iberian Peninsula determined from mitochondrial sequence analysis**. *Ann Hum Genet* 1996, **60**(Pt 4):331-350.

9. Bosch E, Calafell F, Perez-Lezaun A, Clarimon J, Comas D, Mateu E, Martinez-Arias R, Morera B, Brakez Z, Akhayat O *et al*: **Genetic structure of north-west Africa revealed by STR analysis**. *Eur J Hum Genet* 2000, **8**(5):360-366.

10. Merghoub T, Sanchez-Mazas A, Tamouza R, Lu CY, Bouzid K, Ardjoun FZ, Labie D, Lapoumeroulie C, Elion J: **Haemoglobin D-Ouled Rabah among the Mozabites: a relevant variant to trace the origin of Berber-speaking populations**. *Eur J Hum Genet* 1997, **5**(6):390-396.

11. Abu-Amero KK, Larruga JM, Cabrera VM, Gonzalez AM: **Mitochondrial DNA structure in the Arabian Peninsula**. *BMC Evol Biol* 2008, **8**:45.

12. Salem AH, Badr FM, Gaballah MF, Paabo S: **The genetics of traditional living: Y-chromosomal and mitochondrial lineages in the Sinai Peninsula**. *Am J Hum Genet* 1996, **59**(3):741-743.

13. Zlotogora J, Hujerat Y, Barges S, Shalev SA, Chakravarti A: **The fate of 12 recessive mutations in a single village**. *Ann Hum Genet* 2007, **71**(Pt 2):202-208.

14. Richards M, Macaulay V, Hickey E, Vega E, Sykes B, Guida V, Rengo C, Sellitto D, Cruciani F, Kivisild T *et al*: **Tracing European founder lineages in the Near Eastern mtDNA pool**. *Am J Hum Genet* 2000, **67**(5):1251-1276.

15. Shen P, Lavi T, Kivisild T, Chou V, Sengun D, Gefel D, Shpirer I, Woolf E, Hillel J, Feldman MW *et al*: **Reconstruction of patrilineages and matrilineages of Samaritans and other Israeli populations from Y-chromosome and mitochondrial DNA sequence variation**. *Hum Mutat* 2004, **24**(3):248-260.

16. Helgason A, Hickey E, Goodacre S, Bosnes V, Stefansson K, Ward R, Sykes B: **mtDna and the islands of the North Atlantic: estimating the proportions of Norse and Gaelic ancestry**. *Am J Hum Genet* 2001, **68**(3):723-737.

17. Capelli C, Redhead N, Abernethy JK, Gratrix F, Wilson JF, Moen T, Hervig T, Richards M, Stumpf MP, Underhill PA *et al*: **A Y chromosome census of the British Isles**. *Curr Biol* 2003, **13**(11):979-984.

18. Wilson JF, Weiss DA, Richards M, Thomas MG, Bradman N, Goldstein DB: **Genetic evidence for different male and female roles during cultural transitions in the British Isles**. *Proc Natl Acad Sci U S A* 2001, **98**(9):5078-5083.

19. Goodacre S, Helgason A, Nicholson J, Southam L, Ferguson L, Hickey E, Vega E, Stefansson K, Ward R, Sykes B: **Genetic evidence for a family-based Scandinavian settlement of Shetland and Orkney during the Viking periods**. *Heredity* 2005, **95**(2):129-135.

20. Hill WG, Robertson A: **Linkage disequilibrium in finite populations**. *Theoretical and Applied Genetics* 1968, **38**:226-231.

21. Falchi A, Giovannoni L, Calo CM, Piras IS, Moral P, Paoli G, Vona G, Varesi L: **Genetic history of some western Mediterranean human isolates through mtDNA HVR1 polymorphisms**. *J Hum Genet* 2006, **51**(1):9-14.

22. Fraumene C, Belle EM, Castri L, Sanna S, Mancosu G, Cosso M, Marras F, Barbujani G, Pirastu M, Angius A: **High resolution analysis and phylogenetic network construction using complete mtDNA sequences in sardinian genetic isolates**. *Mol Biol Evol* 2006, **23**(11):2101-2111.

23. Semino O, Passarino G, Oefner PJ, Lin AA, Arbuzova S, Beckman LE, De Benedictis G, Francalacci P, Kouvatsi A, Limborska S *et al*: **The genetic legacy of Paleolithic Homo sapiens sapiens in extant Europeans: a Y chromosome perspective**. *Science* 2000, **290**(5494):1155-1159.

24. Angius A, Melis PM, Morelli L, Petretto E, Casu G, Maestrale GB, Fraumene C, Bebbere D, Forabosco P, Pirastu M: **Archival, demographic and genetic studies define a Sardinian sub-isolate as a suitable model for mapping complex traits**. *Hum Genet* 2001, **109**(2):198-209.

25. Service S, DeYoung J, Karayiorgou M, Roos JL, Pretorious H, Bedoya G, Ospina J, Ruiz-Linares A, Macedo A, Palha JA *et al*: **Magnitude and distribution of linkage disequilibrium in population isolates and implications for genome-wide association studies**. *Nat Genet* 2006, **38**(5):556-560.

26. Angius A, Hyland FC, Persico I, Pirastu N, Woodage T, Pirastu M, De la Vega FM: **Patterns of linkage disequilibrium between SNPs in a Sardinian population isolate and the selection of markers for association studies**. *Hum Hered* 2008, **65**(1):9-22.

27. Bertranpetit J, Sala J, Calafell F, Underhill PA, Moral P, Comas D: **Human mitochondrial DNA variation and the origin of Basques**. *Ann Hum Genet* 1995, **59**(Pt 1):63-81.

28. Bosch E, Calafell F, Comas D, Oefner PJ, Underhill PA, Bertranpetit J: **High-resolution analysis of human Y-chromosome variation shows a sharp discontinuity and limited gene flow between northwestern Africa and the Iberian Peninsula**. *Am J Hum Genet* 2001, **68**(4):1019-1029.

29. Calafell F, Bertranpetit J: **Principal component analysis of gene frequencies and the origin of Basques**. *Am J Phys Anthropol* 1994, **93**(2):201-215.

30. Bauduer F, Feingold J, Lacombe D: **The Basques: review of population genetics and Mendelian disorders**. *Hum Biol* 2005, **77**(5):619-637.

31. Rosenberg NA, Pritchard JK, Weber JL, Cann HM, Kidd KK, Zhivotovsky LA, Feldman MW: **Genetic structure of human populations**. *Science* 2002, **298**(5602):2381-2385.

32. Li JZ, Absher DM, Tang H, Southwick AM, Casto AM, Ramachandran S, Cann HM, Barsh GS, Feldman M, Cavalli-Sforza LL *et al*: **Worldwide human relationships inferred from genome-wide patterns of variation**. *Science* 2008, **319**(5866):1100-1104.

33. Quintana-Murci L, Chaix R, Wells RS, Behar DM, Sayar H, Scozzari R, Rengo C, Al-Zahery N, Semino O, Santachiara-Benerecetti AS *et al*: **Where west meets east: the complex mtDNA landscape of the southwest and Central Asian corridor**. *Am J Hum Genet* 2004, **74**(5):827-845.

34. Firasat S, Khaliq S, Mohyuddin A, Papaioannou M, Tyler-Smith C, Underhill PA, Ayub Q: **Y-chromosomal evidence for a limited Greek contribution to the Pathan population of Pakistan**. *Eur J Hum Genet* 2007, **15**(1):121-126.

35. Pakendorf B, Novgorodov IN, Osakovskij VL, Danilova AP, Protod'jakonov AP, Stoneking M: **Investigating the effects of prehistoric migrations in Siberia: genetic variation and the origins of Yakuts**. *Hum Genet* 2006, **120**(3):334-353.

36. Kidd JR, Black FL, Weiss KM, Balazs I, Kidd KK: **Studies of three Amerindian populations using nuclear DNA polymorphisms**. *Hum Biol* 1991, **63**(6):775-794.

37. Calafell F, Shuster A, Speed WC, Kidd JR, Black FL, Kidd KK: **Genealogy reconstruction from short tandem repeat genotypes in an Amazonian population**. *Am J Phys Anthropol* 1999, **108**(2):137-146.
