# Supplementary material for: Decay of linkage disequilibrium within genes across HGDP-CEPH human samples: most population isolates do not show increased LD
Source: BMC Genomics. 2009 Jul 28;10:338. doi: 10.1186/1471-2164-10-338 (PMC2723139; doi:10.1186/1471-2164-10-338)
Supplement: Additional file 3 — List of gene regions used in this study. SNP pairs refers to all possible SNP pairs within each gene region; mean SNP pairs is the average number of SNP pairs actually used per population after dropping those with MAF < 0.05. Abbreviations: CAN, cancer-related genes; GLY, genes involved in glycosylation; IMM, genes related to pathogen recognition and/or immune response; PSY, genes involved in neurotransmission or neurodevelopment; and others, genes belonging to other diverse functional categories. [file 1471-2164-10-338-S3.doc]

**Supplementary Table 1.** List of gene regions used in this study. SNP pairs refers to all possible SNP pairs within each gene region; mean SNP pairs is the average number of SNP pairs actually used per population after dropping those with MAF < 0.05. Abbreviations: CAN, cancer-related genes; GLY, genes involved in glycosylation; IMM, genes related to pathogen recognition and or immune response; PSY, genes involved in neurotransmission or neurodevelopment; and others, genes belonging to other diverse functional categories.

| Gene region | Num  SNPs | DIST (kb) | Mean SNP distance | SNPs/  10 kb | SNP pairs | Mean SNP pairs | Category |
| --- | --- | --- | --- | --- | --- | --- | --- |
| *ADPRT* | 7 | 47.84 | 7.97 | 1.46 | 21 | 17.36 | CAN |
| *AGTR1* | 6 | 53.84 | 10.77 | 1.11 | 15 | 6.62 | CAN |
| *AKT1* | 3 | 35.13 | 17.57 | 0.85 | 3 | 2.79 | CAN |
| *ALDH2_MAPKAPK5* | 10 | 144.87 | 16.10 | 0.69 | 45 | 30.13 | CAN |
| *APAF1* | 4 | 73.71 | 24.57 | 0.54 | 6 | 4.77 | CAN |
| *APC* | 7 | 100.84 | 16.81 | 0.69 | 21 | 15.64 | CAN |
| *APEX1* | 3 | 6.89 | 3.44 | 4.35 | 3 | 2.90 | CAN |
| *ARHGDIB* | 6 | 28.31 | 5.66 | 2.12 | 15 | 14.87 | CAN |
| *ATM* | 11 | 144.41 | 14.44 | 0.76 | 55 | 37.85 | CAN |
| *BAX* | 2 | 2.74 | 2.74 | 7.31 | 1 | 0.77 | CAN |
| *BCL2* | 17 | 191.23 | 11.95 | 0.89 | 136 | 109.21 | CAN |
| *BCL2L1* | 4 | 67.86 | 22.62 | 0.59 | 6 | 3.56 | CAN |
| *BCL6* | 8 | 26.13 | 3.73 | 3.06 | 28 | 23.51 | CAN |
| *BCR* | 8 | 111.57 | 15.94 | 0.72 | 28 | 22.85 | CAN |
| *BLM* | 7 | 63.97 | 10.66 | 1.09 | 21 | 18.54 | CAN |
| *BRAF* | 5 | 191.90 | 47.97 | 0.26 | 10 | 8.28 | CAN |
| *BRCA1* | 6 | 80.84 | 16.17 | 0.74 | 15 | 11.51 | CAN |
| *BRCA2* | 9 | 84.53 | 10.57 | 1.06 | 36 | 28.03 | CAN |
| *BRMS1* | 3 | 37.54 | 18.77 | 0.80 | 3 | 2.64 | CAN |
| *BUB1B* | 5 | 68.12 | 17.03 | 0.73 | 10 | 10.00 | CAN |
| *CASP3* | 3 | 23.77 | 11.89 | 1.26 | 3 | 1.64 | CAN |
| *CASP8_CASP10* | 9 | 102.55 | 12.82 | 0.88 | 36 | 31.28 | CAN |
| *CASP9* | 3 | 8.07 | 4.03 | 3.72 | 3 | 2.38 | CAN |
| *CCNA2* | 2 | 1.33 | 1.33 | 15.00 | 1 | 0.95 | CAN |
| *CCND3* | 4 | 13.05 | 4.35 | 3.07 | 6 | 4.44 | CAN |
| *CD44* | 11 | 97.44 | 9.74 | 1.13 | 55 | 39.26 | CAN |
| *CDC25A* | 5 | 36.43 | 9.11 | 1.37 | 10 | 5.87 | CAN |
| *CDK2* | 2 | 3.45 | 3.45 | 5.81 | 1 | 0.59 | CAN |
| *CDK4* | 2 | 2.30 | 2.30 | 8.69 | 1 | 0.64 | CAN |
| *CDK6* | 18 | 235.26 | 13.84 | 0.77 | 153 | 104.97 | CAN |
| *CDKN2A_CDKN2B* | 10 | 51.62 | 5.74 | 1.94 | 45 | 25.28 | CAN |
| *CDKN2C* | 3 | 11.75 | 5.88 | 2.55 | 3 | 2.03 | CAN |
| *CLDN8* | 9 | 53.10 | 6.64 | 1.69 | 36 | 21.18 | CAN |
| *CRSP3* | 4 | 47.43 | 15.81 | 0.84 | 6 | 5.31 | CAN |
| *CYP17A1* | 3 | 14.18 | 7.09 | 2.12 | 3 | 2.33 | CAN |
| *CYP1A1* | 3 | 9.80 | 4.90 | 3.06 | 3 | 2.38 | CAN |
| *CYP2A7* | 15 | 183.55 | 13.11 | 0.82 | 105 | 95.44 | CAN |
| *CYP2D6* | 2 | 37.48 | 37.48 | 0.53 | 1 | 0.79 | CAN |
| *CYP2E1* | 3 | 10.80 | 5.40 | 2.78 | 3 | 2.51 | CAN |
| *CYPIIB1_CYPIIB2* | 6 | 47.75 | 9.55 | 1.26 | 15 | 11.82 | CAN |
| *DACT1* | 12 | 72.01 | 6.55 | 1.67 | 66 | 22.23 | CAN |
| *DIAPH1* | 24 | 178.16 | 7.75 | 1.35 | 276 | 90.69 | CAN |
| *E2F1* | 3 | 9.24 | 4.62 | 3.25 | 3 | 2.69 | CAN |
| *E2F3* | 4 | 84.08 | 28.03 | 0.48 | 6 | 5.03 | CAN |
| *EGF* | 11 | 100.91 | 10.09 | 1.09 | 55 | 43.90 | CAN |
| *EGFR* | 18 | 187.48 | 11.03 | 0.96 | 153 | 98.79 | CAN |
| *EPHX1* | 4 | 32.51 | 10.84 | 1.23 | 6 | 5.38 | CAN |
| *ERBB2* | 3 | 18.03 | 9.02 | 1.66 | 3 | 2.59 | CAN |
| *ERCC1_ERCC2* | 10 | 76.30 | 8.48 | 1.31 | 45 | 38.97 | CAN |
| *ERCC4* | 5 | 26.88 | 6.72 | 1.86 | 10 | 8.85 | CAN |
| *ERCC6* | 12 | 74.95 | 6.81 | 1.60 | 66 | 47.82 | CAN |
| *FANCA* | 8 | 79.56 | 11.37 | 1.01 | 28 | 22.08 | CAN |
| *FOS* | 3 | 5.51 | 2.75 | 5.45 | 3 | 2.00 | CAN |
| *GRB2* | 4 | 44.08 | 14.69 | 0.91 | 6 | 4.62 | CAN |
| *GSTP1* | 6 | 8.39 | 1.68 | 7.15 | 15 | 7.44 | CAN |
| *HDAC2* | 5 | 40.30 | 10.07 | 1.24 | 10 | 7.90 | CAN |
| *HIF1A* | 5 | 47.29 | 11.82 | 1.06 | 10 | 7.18 | CAN |
| *HPSE* | 4 | 36.81 | 12.27 | 1.09 | 6 | 4.90 | CAN |
| *IL1A* | 4 | 16.52 | 5.51 | 2.42 | 6 | 5.85 | CAN |
| *IL2* | 3 | 10.63 | 5.31 | 2.82 | 3 | 2.77 | CAN |
| *KAI1* | 3 | 20.75 | 10.38 | 1.45 | 3 | 2.36 | CAN |
| *LIG1* | 9 | 57.11 | 7.14 | 1.58 | 36 | 32.79 | CAN |
| *LIG3* | 6 | 17.85 | 3.57 | 3.36 | 15 | 8.10 | CAN |
| *LIG4* | 4 | 13.26 | 4.42 | 3.02 | 6 | 1.74 | CAN |
| *MAD2L1* | 4 | 21.73 | 7.24 | 1.84 | 6 | 5.62 | CAN |
| *MAP2K4* | 10 | 116.56 | 12.95 | 0.86 | 45 | 35.08 | CAN |
| *MAP2K6* | 8 | 50.46 | 7.21 | 1.59 | 28 | 24.51 | CAN |
| *MAPK14* | 9 | 94.35 | 11.79 | 0.95 | 36 | 14.90 | CAN |
| *MAPKAPK2* | 4 | 42.49 | 14.16 | 0.94 | 6 | 5.08 | CAN |
| *MDM2* | 6 | 32.24 | 6.45 | 1.86 | 15 | 8.54 | CAN |
| *MLH1* | 4 | 64.41 | 21.47 | 0.62 | 6 | 5.46 | CAN |
| *MMP2* | 9 | 43.28 | 5.41 | 2.08 | 36 | 30.74 | CAN |
| *MMP3* | 4 | 9.02 | 3.01 | 4.44 | 6 | 4.77 | CAN |
| *MSH2* | 9 | 85.31 | 10.66 | 1.06 | 36 | 31.36 | CAN |
| *MSH3* | 18 | 230.37 | 13.55 | 0.78 | 153 | 119.85 | CAN |
| *MSH6* | 4 | 21.02 | 7.01 | 1.90 | 6 | 3.56 | CAN |
| *MTCBP-1* | 21 | 1928.15 | 96.41 | 0.11 | 210 | 189.00 | CAN |
| *MTHFR* | 3 | 4.08 | 2.04 | 7.36 | 3 | 2.62 | CAN |
| *NAT1* | 4 | 13.35 | 4.45 | 3.00 | 6 | 3.28 | CAN |
| *NAT2* | 6 | 15.84 | 3.17 | 3.79 | 15 | 11.77 | CAN |
| *NFKB1* | 11 | 123.06 | 12.31 | 0.89 | 55 | 45.72 | CAN |
| *NFKBIA* | 4 | 9.39 | 3.13 | 4.26 | 6 | 4.95 | CAN |
| *NM_134434* | 12 | 115.17 | 10.47 | 1.04 | 66 | 62.18 | CAN |
| *NME1* | 2 | 1.96 | 1.96 | 10.22 | 1 | 0.97 | CAN |
| *NME4* | 2 | 0.45 | 0.45 | 44.44 | 1 | 1.00 | CAN |
| *PCNA* | 3 | 4.29 | 2.15 | 6.99 | 3 | 1.72 | CAN |
| *PIK3CB* | 9 | 115.63 | 14.45 | 0.78 | 36 | 23.49 | CAN |
| *PIK3R1* | 11 | 80.49 | 8.05 | 1.37 | 55 | 42.62 | CAN |
| *PIK3R2* | 2 | 5.57 | 5.57 | 3.59 | 1 | 1.00 | CAN |
| *PTEN* | 7 | 115.39 | 19.23 | 0.61 | 21 | 16.59 | CAN |
| *PTTG1* | 4 | 17.21 | 5.74 | 2.32 | 6 | 4.31 | CAN |
| *RAD54L* | 2 | 4.80 | 4.80 | 4.17 | 1 | 0.74 | CAN |
| *RB1* | 13 | 180.70 | 15.06 | 0.72 | 78 | 42.54 | CAN |
| *RECQL* | 6 | 26.85 | 5.37 | 2.23 | 15 | 10.77 | CAN |
| *RELA* | 4 | 8.77 | 2.92 | 4.56 | 6 | 5.36 | CAN |
| *RET* | 9 | 65.19 | 8.15 | 1.38 | 36 | 29.79 | CAN |
| *SOD2* | 4 | 29.24 | 9.75 | 1.37 | 6 | 5.59 | CAN |
| *SOS1* | 3 | 87.42 | 43.71 | 0.34 | 3 | 2.36 | CAN |
| *STAT1* | 4 | 44.80 | 14.93 | 0.89 | 6 | 5.13 | CAN |
| *STK6* | 4 | 24.08 | 8.03 | 1.66 | 6 | 5.00 | CAN |
| *TERT* | 3 | 34.80 | 17.40 | 0.86 | 3 | 2.18 | CAN |
| *TMPRSS2* | 19 | 112.22 | 6.23 | 1.69 | 171 | 126.74 | CAN |
| *TNFRSF6* | 6 | 36.02 | 7.20 | 1.67 | 15 | 9.85 | CAN |
| *TNFSF10* | 4 | 17.56 | 5.85 | 2.28 | 6 | 2.13 | CAN |
| *TNFSF6* | 3 | 17.25 | 8.63 | 1.74 | 3 | 2.38 | CAN |
| *TP73L* | 18 | 262.28 | 15.43 | 0.69 | 153 | 136.85 | CAN |
| *TRAF6* | 2 | 13.07 | 13.07 | 1.53 | 1 | 0.62 | CAN |
| *USP2* | 22 | 400.59 | 19.08 | 0.55 | 231 | 156.90 | CAN |
| *VEGF* | 7 | 27.25 | 4.54 | 2.57 | 21 | 18.38 | CAN |
| *WRN* | 11 | 118.37 | 11.84 | 0.93 | 55 | 39.77 | CAN |
| *XPA* | 3 | 28.29 | 14.15 | 1.06 | 3 | 1.72 | CAN |
| *XPC* | 7 | 27.52 | 4.59 | 2.54 | 21 | 14.38 | CAN |
| *XRCC1* | 7 | 32.15 | 5.36 | 2.18 | 21 | 15.21 | CAN |
| *XRCC2* | 5 | 28.13 | 7.03 | 1.78 | 10 | 5.46 | CAN |
| *XRCC3* | 2 | 7.05 | 7.05 | 2.84 | 1 | 0.82 | CAN |
| *XRCC4* | 16 | 264.23 | 17.62 | 0.61 | 120 | 101.69 | CAN |
| *C1GALT1* | 4 | 33.95 | 11.32 | 1.18 | 6 | 4.85 | GLY |
| *FUT1* | 17 | 122.00 | 7.63 | 1.39 | 136 | 104.67 | GLY |
| *FUT10* | 14 | 162.71 | 12.52 | 0.86 | 91 | 68.90 | GLY |
| *FUT11* | 8 | 64.82 | 9.26 | 1.23 | 28 | 23.31 | GLY |
| *FUT4* | 8 | 58.23 | 8.32 | 1.37 | 28 | 22.23 | GLY |
| *FUT5* | 10 | 118.02 | 13.11 | 0.85 | 45 | 33.46 | GLY |
| *FUT8* | 29 | 395.41 | 14.12 | 0.73 | 406 | 293.41 | GLY |
| *FUT9* | 25 | 250.85 | 10.45 | 1.00 | 300 | 213.44 | GLY |
| *GALNT1* | 15 | 114.93 | 8.21 | 1.31 | 105 | 78.08 | GLY |
| *GALNT10* | 38 | 293.63 | 7.94 | 1.29 | 703 | 469.28 | GLY |
| *GALNT12* | 12 | 110.15 | 10.01 | 1.09 | 66 | 57.23 | GLY |
| *GALNT13* | 46 | 567.08 | 12.60 | 0.81 | 1035 | 755.77 | GLY |
| *GALNT14* | 28 | 288.17 | 10.67 | 0.97 | 378 | 321.15 | GLY |
| *GALNT15* | 11 | 93.81 | 9.38 | 1.17 | 55 | 46.62 | GLY |
| *GALNT2* | 25 | 256.99 | 10.71 | 0.97 | 300 | 225.13 | GLY |
| *GALNT3* | 9 | 75.34 | 9.42 | 1.19 | 36 | 32.41 | GLY |
| *GALNT4* | 10 | 53.84 | 5.98 | 1.86 | 45 | 29.54 | GLY |
| *GALNT5* | 7 | 83.20 | 13.87 | 0.84 | 21 | 11.49 | GLY |
| *GALNT6* | 15 | 80.95 | 5.78 | 1.85 | 105 | 79.36 | GLY |
| *GALNT8* | 12 | 116.46 | 10.59 | 1.03 | 66 | 35.38 | GLY |
| *GALNTL1* | 21 | 154.34 | 7.72 | 1.36 | 210 | 117.05 | GLY |
| *GCNT1* | 8 | 107.92 | 15.42 | 0.74 | 28 | 24.18 | GLY |
| *GCNT3* | 5 | 63.54 | 15.88 | 0.79 | 10 | 8.49 | GLY |
| *GLTDC1* | 9 | 69.19 | 8.65 | 1.30 | 36 | 20.31 | GLY |
| *HDHD3* | 19 | 430.07 | 23.89 | 0.44 | 171 | 130.74 | GLY |
| *SIAT1* | 15 | 193.50 | 13.82 | 0.78 | 105 | 88.08 | GLY |
| *SIAT10* | 13 | 90.33 | 7.53 | 1.44 | 78 | 70.67 | GLY |
| *SIAT4A* | 22 | 170.85 | 8.14 | 1.29 | 231 | 180.00 | GLY |
| *SIAT4C* | 12 | 118.85 | 10.80 | 1.01 | 66 | 45.54 | GLY |
| *SIAT6* | 26 | 280.90 | 11.24 | 0.93 | 325 | 273.64 | GLY |
| *SIAT7A* | 9 | 70.84 | 8.86 | 1.27 | 36 | 24.26 | GLY |
| *SIAT7B* | 10 | 84.60 | 9.40 | 1.18 | 45 | 22.36 | GLY |
| *SIAT7C* | 57 | 616.50 | 11.01 | 0.92 | 1596 | 1237.82 | GLY |
| *SIAT7D* | 3 | 36.55 | 18.27 | 0.82 | 3 | 2.44 | GLY |
| *SIAT7E* | 21 | 263.15 | 13.16 | 0.80 | 210 | 145.59 | GLY |
| *SIAT8A* | 17 | 146.66 | 9.17 | 1.16 | 136 | 103.13 | GLY |
| *SIAT8B* | 17 | 127.91 | 7.99 | 1.33 | 136 | 104.28 | GLY |
| *SIAT8C* | 14 | 69.04 | 5.31 | 2.03 | 91 | 45.54 | GLY |
| *SIAT8D* | 15 | 116.07 | 8.29 | 1.29 | 105 | 66.13 | GLY |
| *SIAT8E* | 14 | 135.91 | 10.45 | 1.03 | 91 | 67.49 | GLY |
| *SIAT9* | 12 | 107.60 | 9.78 | 1.12 | 66 | 50.69 | GLY |
| *ST6GALNAC6* | 4 | 31.67 | 10.56 | 1.26 | 6 | 2.95 | GLY |
| *Transferase* | 9 | 73.18 | 9.15 | 1.23 | 36 | 31.62 | GLY |
| *AIRE* | 15 | 84.01 | 6.00 | 1.79 | 105 | 76.74 | IMM |
| *CD14* | 8 | 63.97 | 9.14 | 1.25 | 28 | 25.67 | IMM |
| *CDs* | 15 | 244.10 | 17.44 | 0.61 | 105 | 64.23 | IMM |
| *DC-SIGN* | 7 | 62.62 | 10.44 | 1.12 | 21 | 16.08 | IMM |
| *Il10* | 9 | 62.53 | 7.82 | 1.44 | 36 | 25.18 | IMM |
| *IL15RA* | 21 | 90.06 | 4.50 | 2.33 | 210 | 37.67 | IMM |
| *IL1RL2* | 22 | 119.26 | 5.68 | 1.84 | 231 | 155.56 | IMM |
| *Il6* | 9 | 53.89 | 6.74 | 1.67 | 36 | 15.59 | IMM |
| *MBL2* | 9 | 56.85 | 7.11 | 1.58 | 36 | 24.28 | IMM |
| *MRC2* | 23 | 418.19 | 19.01 | 0.55 | 253 | 196.72 | IMM |
| *PTGER4* | 20 | 395.25 | 20.80 | 0.51 | 190 | 175.44 | IMM |
| *TLR4* | 11 | 76.13 | 7.61 | 1.44 | 55 | 21.26 | IMM |
| *TLR9* | 9 | 51.89 | 6.49 | 1.73 | 36 | 31.05 | IMM |
| *TLRs* | 22 | 120.28 | 5.73 | 1.83 | 231 | 160.72 | IMM |
| *VPS37C* | 22 | 414.97 | 19.76 | 0.53 | 231 | 145.10 | IMM |
| *DBH* | 16 | 72.23 | 4.82 | 2.22 | 120 | 66.95 | PSY |
| *DDC* | 21 | 154.89 | 7.74 | 1.36 | 210 | 158.62 | PSY |
| *DRD1* | 8 | 58.80 | 8.40 | 1.36 | 28 | 18.67 | PSY |
| *DRD2* | 16 | 111.06 | 7.40 | 1.44 | 120 | 96.08 | PSY |
| *DRD3* | 14 | 104.12 | 8.01 | 1.34 | 91 | 75.36 | PSY |
| *DRD4* | 6 | 41.81 | 8.36 | 1.44 | 15 | 12.38 | PSY |
| *DRD5* | 7 | 48.58 | 8.10 | 1.44 | 21 | 15.56 | PSY |
| *GFRA3* | 20 | 395.64 | 20.82 | 0.51 | 190 | 138.77 | PSY |
| *HTR1A* | 6 | 49.58 | 9.92 | 1.21 | 15 | 14.49 | PSY |
| *HTR1B* | 10 | 57.26 | 6.36 | 1.75 | 45 | 21.90 | PSY |
| *HTR2A* | 18 | 117.82 | 6.93 | 1.53 | 153 | 107.46 | PSY |
| *HTR4* | 32 | 261.47 | 8.43 | 1.22 | 496 | 393.18 | PSY |
| *NRG1* | 45 | 1168.49 | 26.56 | 0.39 | 990 | 779.08 | PSY |
| *PPP1R1B* | 7 | 59.52 | 9.92 | 1.18 | 21 | 14.08 | PSY |
| *RELN* | 69 | 578.50 | 8.51 | 1.19 | 2346 | 1462.10 | PSY |
| *SLC6A3* | 16 | 92.13 | 6.14 | 1.74 | 120 | 94.08 | PSY |
| *SLC6A4* | 14 | 94.10 | 7.24 | 1.49 | 91 | 68.49 | PSY |
| *TH* | 9 | 62.92 | 7.86 | 1.43 | 36 | 29.33 | PSY |
| *TPH1* | 9 | 80.10 | 10.01 | 1.12 | 36 | 27.90 | PSY |
| *TPH2* | 17 | 122.70 | 7.67 | 1.39 | 136 | 108.64 | PSY |
| *COMT* | 16 | 73.37 | 4.89 | 2.18 | 120 | 103.79 | PSY |
| *ALDH6A1* | 15 | 89.84 | 6.42 | 1.67 | 105 | 51.03 | Other |
| *BCKDHA* | 17 | 84.54 | 5.28 | 2.01 | 136 | 90.18 | Other |
| *CA14* | 17 | 430.31 | 26.89 | 0.40 | 136 | 96.05 | Other |
| *CST2* | 9 | 59.42 | 7.43 | 1.51 | 36 | 25.77 | Other |
| *EYA4* | 41 | 362.70 | 9.07 | 1.13 | 820 | 582.62 | Other |
| *FOXI1* | 21 | 382.38 | 19.12 | 0.55 | 210 | 145.18 | Other |
| *GIP* | 9 | 77.40 | 9.67 | 1.16 | 36 | 24.38 | Other |
| *GSTZ1* | 20 | 81.16 | 4.27 | 2.46 | 190 | 107.56 | Other |
| *HCLS1* | 14 | 95.90 | 7.38 | 1.46 | 91 | 50.44 | Other |
| *LCT* | 24 | 149.72 | 6.51 | 1.60 | 276 | 162.26 | Other |
| *LHPP* | 26 | 457.71 | 18.31 | 0.57 | 325 | 272.05 | Other |
| *MRPL35* | 13 | 77.02 | 6.42 | 1.69 | 78 | 33.03 | Other |
| *OR2A14* | 16 | 470.32 | 31.35 | 0.34 | 120 | 104.56 | Other |
| *OR5G1P* | 17 | 350.09 | 21.88 | 0.49 | 136 | 114.49 | Other |
| *OR5I1* | 11 | 58.42 | 5.84 | 1.88 | 55 | 15.21 | Other |
| *TECTA* | 25 | 152.96 | 6.37 | 1.63 | 300 | 163.44 | Other |
